# Elevated Levels of Interferon-*γ* Are Associated with High Levels of Epstein-Barr Virus Reactivation in Patients with the Intestinal Type of Gastric Cancer

**DOI:** 10.1155/2017/7069242

**Published:** 2017-11-14

**Authors:** María G. Cárdenas-Mondragón, Javier Torres, Norma Sánchez-Zauco, Alejandro Gómez-Delgado, Margarita Camorlinga-Ponce, Carmen Maldonado-Bernal, Ezequiel M. Fuentes-Pananá

**Affiliations:** ^1^Unidad de Investigación Médica en Enfermedades Infecciosas y Parasitarias (UIMEIP), Hospital de Pediatría, Dr. Silvestre Frenk Freund, CMN Siglo-XXI, Instituto Mexicano del Seguro Social (IMSS), Ciudad de México, Mexico; ^2^Laboratorio de Investigación en Inmunología y Proteómica, Hospital Infantil de México “Federico Gómez”, Ciudad de México, Mexico; ^3^Unidad de Investigación en Virología y Cáncer, Hospital Infantil de México “Federico Gómez”, Ciudad de México, Mexico

## Abstract

**Background:**

The inflammatory response directed against *Helicobacter pylori* (*HP*) is believed to be one of the main triggers of the appearance of gastric lesions and their progression to gastric cancer (GC). Epstein-Barr virus (EBV) has been found responsible for about 10% of all GCs, but the inflammatory response has not been studied in GC patients with evidence of high levels of EBV reactivation.

**Objective:**

To determine the relationship between inflammation and antibodies against EBV reactivation antigens, *HP*, and the bacterium virulence factor CagA in patients with GC.

**Methods:**

127 GC patients, 46 gastritis patients, and 197 healthy subjects were studied. IL-1*β*, IL-6, IL-8, IL-10, TNF-*α*, TGF-*β*, MCP-1, and IFN-*γ* levels were measured in serum or plasma and compared against the antibody titers of VCA-IgG, *HP*, and the *HP* virulence factor CagA. Statistical associations were estimated.

**Results:**

Significant ORs and positive trends were found between VCA-IgG and IFN-*γ*, specifically for patients with GC of intestinal type (OR: 6.4, 95% C.I. 1.2–35.4) (*p* < 0.044).

**Conclusions:**

We confirmed a positive association between a marker of EBV reactivation and intestinal gastric cancer and present evidence of a correlation with elevated serum levels of IFN-*γ*, but not with the other cytokines.

## 1. Introduction

Approximately 952,000 new cases of gastric cancer (GC) were estimated in 2012 in the world, and with 723,000 deaths, GC is the third cause of death from cancer worldwide [1, 2]. According to Laurent's classification, GC can be divided into two subtypes: intestinal and diffuse [3]. GC is considered an inflammatory disease because it evolves through a series of progressive inflammatory lesions. Both the intestinal and diffuse types of GC originate within a chronic inflammatory gastritis, in which the intestinal type evolves into atrophic gastritis, intestinal metaplasia, dysplasia, and GC [4, 5]. Progression to the diffuse type does not seem to follow the intermediate inflammatory lesion route, and it has been associated with mutations in adhesion proteins, such as E-cadherin [6].

GC is thought to have an infectious origin, with *Helicobacter pylori* (*HP*) and Epstein-Barr virus (EBV) considered the main associated pathogens [7–10]. *HP* is considered the main risk factor to develop GC, with most studies supporting a direct transforming role for EBV in only about 10% of cases. In agreement, The Cancer Genome Atlas (TCGA), based on the high number of viral copies and high levels of viral gene expression, proposed the EBV-positive GC (also known as EBVaGC) as a particular GC entity characterized by DNA hypermethylation, increased frequency of *PIK3CA*, *ARID1A*, and *BCOR* mutations but wild-type *p53*, and high expression of T cell inhibitory molecules *CD274* (*PD-L1*), *PDCD1LG2* (*PD-L2*), and *indoleamine 2,3-dioxygenase 1* (*IDO 1*) [10, 11].

Since *HP* is the persistent resident of the gastric mucosa, the inflammatory response directed against the bacteria is considered the main trigger of the chronic inflammatory response leading to GC. On the other hand, EBV is a lymphotropic virus that is thought to persist in memory B cells, probably in secondary lymph nodes and bone marrow. How and when EBV reaches the stomach is presently unknown. Because the virus is transmitted by saliva and virus shedding is detected in the lymph nodes of the oropharynx, it is thought that the virus is constantly reactivating in this area. In agreement, EBV is associated with nasopharyngeal carcinoma (NPC) and increased levels of antibodies directed against viral reactivation antigens, such as late lytic structural proteins, marks individuals with an increased risk to develop NPC [12–16]. We have recently made a similar observation for gastric cancer, finding a positive association between high levels of antibodies against the viral capsid antigen (VCA) and an increased risk to develop intestinal GC in adults and increased risk to develop severe gastritis in children [17, 18]. Patients exhibiting the highest levels of antibodies against both EBV and *HP* also presented the highest levels of immune cell infiltration and risk to develop a severe inflammatory disease, supporting a cofactor role for EBV as a trigger of gastric inflammation together with *HP*. In this study, in a new prospective cohort of GC patients, we evaluated VCA-IgG, which we have previously found closely correlating with increased inflammatory gastric infiltrate and denoting increased risk to develop gastric cancer. We also evaluated the serum levels of eight mediators of inflammation: interleukin-1beta (IL-1*β*), interleukin-6 (IL-6), interleukin-8 (IL-8), interleukin-10 (IL-10), tumor necrosis factor-alpha (TNF-*α*), transforming growth factor-beta (TGF-*β*), monocyte chemoattractant protein-1 (MCP-1/CCL2), and interferon-gamma (IFN-*γ*). These cytokines are known to mediate gastric damage or immune antipathogen responses. We found that increased levels of VCA-IgG antibodies are significantly associated with elevated serum levels of IFN-*γ*, particularly in intestinal type of GC.

## 2. Materials and Methods

### 2.1. Ethics Statement

The Scientific and Ethics Committees from each of the participating hospitals approved this study: Hospital de Oncologia and Hospital de Especialidades of Centro Médico Nacional siglo XXI (Instituto Mexicano del Seguro Social; IMSS), Instituto Nacional de Cancerología (Secretaría de Salud), Hospital General de México “Eduardo Liceaga” (Secretaría de Salud), all these hospitals in México City, and the Blood Bank of the Centro Médico Nacional Siglo XXI-IMSS in México City. All patients and healthy individuals (HI) were informed on the nature of the study, and those willing to participate signed a written informed consent prior to specimen collection.

### 2.2. Study Design

This is an analytic cross-sectional study of patients with GC in which antibodies against: the EBV reactivation antigen VCA, *HP*, and the *HP* CagA virulence factor were measured in serum or plasma and analyzed for possible associations with the type of GC and the levels of eight cytokines: IL-1*β*, IL-6, IL-8, IL-10, TNF-*α*, TGF-*β*, MCP-1, and IFN-*γ*.

### 2.3. Study Population

The study included 127 GC patients. In an initial study, IL-8, IFN-*γ*, and TGF-*β* were analyzed in all the GC patients and compared with 118 HI. This study was later extended to include IL-1*β*, TNF-*α*, IL-6, IL-10, and MCP-1; this latter analysis was performed in 65 GC patients and 113 HI (Supplementary Table S1 available online at https://doi.org/10.1155/2017/7069242). The HI samples were taken from a bank of sera and plasma of 197 HI. We also analyzed 46 patients with nonatrophic gastritis. In total 370 adult individuals were analyzed: 127 GC, 46 gastritis patients, and 197 HI.

### 2.4. Data Collected

Socio-demographic data and clinical information were registered in questionnaires at the time of inclusion. The information collected included age, gender, and clinical symptoms. Disease diagnosis was made based on clinical presentation, endoscopy, and histological analysis of a biopsy, and GC classification was based on the histology of the resected tumor. Patients treated with antibiotics, bismuth compounds, proton pump inhibitors, nonsteroidal anti-inflammatory drugs, antiacids, or chemotherapy three weeks prior to sample collection were excluded from the study.

### 2.5. Clinical and Histopathological Analysis

Patients were subjected to endoscopy, and six gastric biopsies were taken for an initial diagnosis; the surgically resected tumor was used for confirmatory diagnosis. Biopsies and tumor samples were fixed in formalin, embedded in paraffin, and a section stained with hematoxylin and eosin (HE). HE-stained sections were used to measure and classify the histological type of gastric adenocarcinoma: intestinal, diffuse, or mixed [19]. An expert pathologist analyzed all samples after standardization of the criteria using consensus protocol reading.

### 2.6. Collection of Blood

A sample of venous blood (4 ml) was drawn from all patients. Sera or plasma was collected and stored at −20°C until analysis. Stored samples were used to analyze antibodies and cytokines.

### 2.7. Determination of Anti-EBV-VCA Antibodies

VCA antibodies were determined using ELISA commercial kits (HUMAN; Wiesbaden, Germany), for IgG anti-VCA (catalog 51204) following manufacturer instructions and as previously described [17, 18]. The reported value is the average of two independent assays. A subgroup of samples was done in quadruplicate using different lots of the ELISA kits to check for reproducibility. Calculations for antibody titers were done according to the manufacturer's instructions, and the values are reported as HU units/ml for IgG.

### 2.8. Determination of Antibodies Anti-Whole *H. pylori* Extracts and Anti-CagA

IgG antibodies against *HP* and CagA were determined using ELISA tests previously used and validated in a Mexican population [20]. Patients were considered positive for *HP* antibodies when ELISA units were ≥1.0, and for CagA when ELISA units were ≥1.5, according to the validated cut-offs.

### 2.9. Determination of Mediators of Inflammation

The concentration of IL-8, IFN-*γ*, and TGF-*β* in plasma samples was measured by ELISA using commercially available kits (BD™OptEIA; BD Biosciences, Rockville, MD) according to the manufacturer's instructions. The IL-1*β*, IL-6, TNF-*α*, IL-10, and MCP-1 levels were measured in serum using a specifically designed Multiplex Immunoassay Milliplex® MAP Kit (Merck Millipore, Darmstadt, Germany) Cat. HCYTOMAG-60K-HUM and Magpix Luminex Milliplex ® Analyzer for xMAP ® technology, following the manufacturer's recommended protocols. Estimated cut-off values and areas under the curve for each cytokine and chemokine using ROC analyses were determined according to Sánchez-Zauco et al. [21]. These cytokine results were obtained from a previous study with the same set of samples.

### 2.10. Detection of EBV Sequences

DNA samples were subjected to a first PCR with primers LLW1 and LLW2 and nested PCR by LLWint2 and LLWint2 as previously described [22]. Briefly, the PCR mix (50 *μ*l) contained 200 ng of template DNA, 200 *μ*M of dNTPs mix, 2.5 mM of MgCl_2_, 5 *μ*l of Taq polymerase buffer 10x with (NH_4_)_2_SO_4_, 200 nM of each primer, and 2.5 U of Taq polymerase (all from Thermo Fisher Scientific). The PCR reaction was an initial denaturation step of 5 min at 94°C and then 30 cycles of 94°C for 1.5 min, 57°C for 45 s and 72°C for 1 min, and a final extension of 72°C for 7 min. The nested PCR contained a 50 *μ*l PCR mixture: 1 *μ*l of the first PCR (1 : 1000 final dilution), 200 *μ*M of dNTPs mix, 2.5 mM of MgCl_2_, 5 *μ*l of Taq polymerase buffer 10x with (NH_4_)_2_SO_4_, 400 nM of each primer, and 2.5 U of Taq Polymerase. The reaction was performed with an initial denaturation step at 94°C for 5 min, followed by 15 cycles of 94°C for 20 s, 57°C for 20 s, and 72°C for 30 s, and a final extension of 72°C for 7 min. DNA from Daudi cells an EBV-positive cell line was used as positive control. The PCR products of positive samples were purified using QIAquick gel extraction kit (Qiagen) according to manufacturer's instructions, and forward and reverse strands were sequenced at the Biology Institute, National Autonomous University of Mexico. To confirm identity with EBV, sequences were compared with the GenBank database using the BLAST program.

### 2.11. Statistical Analysis

The dataset was analyzed using different statistical tests. For continuous variables with normal distribution, the mean and standard deviation were used; if the variable did not show a normal distribution, the median and range were used. Nonnormally distributed variables (antibody titers) were analyzed by the Kruskal-Wallis followed by the Mann–Whitney *U* tests. A one-way analysis of variance (ANOVA) followed by the Student *t-*test was used to address age differences. Associations between the type of GC and anti-VCA-IgG titers were estimated using odds ratios (ORs) with 95% confidence intervals (CIs). For this analysis, the VCA-IgG titer was categorized by tertiles based on their distribution in the HI control group, followed by a comparison of the highest to the lowest tertiles. A test for trend was used to analyze whether increased IgG titers are associated with increased ORs. ORs were also used to estimate the association between cytokines and the frequency of EBV and *HP* positives (coinfection) in GC. Because sex and age are confounders, some analyses were adjusted for these two variables.

## 3. Results

### 3.1. Study Population

The study included 127 adult patients with GC, 46 with nonatrophic gastritis, and 197 healthy blood donors that were incorporated as controls (termed as healthy individuals or HI). EBV, HP and CagA antibodies (IgG), and cytokines were measured in plasma taken from GC patients and controls. We first compared the GC patients with a subgroup of the eldest 118 HI controls. Age, gender, and the anti-EBV-VCA titer of patients and HI controls are summarized in Table 1. Similar results were found using the total HI (*n* = 197, Supplementary Table S2). Because blood donors tend to be young volunteers, the HI control group was significantly younger than patients in all groups of GC. Therefore, all the analyses performed were adjusted by age and gender. The GC group was classified according to Laurent's as follows: 40 (31.5%) cases were intestinal type, 63 (49.6%) were diffuse, and 24 (18.9%) were mixed.

### 3.2. Association between EBV-VCA Antibodies and Gastric Cancer

We have previously reported an association between VCA-IgG antibodies and the intestinal type of GC with a different cohort of patients [18]. We started with a similar analysis to the one previously published, VCA-IgG titers were categorized by tertiles based on their distribution in the HI control group, and odds ratios and trends were estimated between GC and each group of increasing anti-EBV-VCA antibody titers (Table 1 and Supplementary Table 2). Although this cohort includes a significant lesser number of patients than our initial study, we confirmed a positive association with the intestinal type of GC. No other significant associations were found. The association between VCA-IgG titers and intestinal type of GC was not related to age, as the age distribution of patients was similar between all the tertiles (Supplementary Figure 1).

### 3.3. Association between Inflammatory Mediators and Anti-VCA-IgG Antibodies

A combined analysis of GC, anti-VCA-IgG antibodies, and eight inflammatory mediators was performed to better understand the role of EBV in gastric chronic inflammation that characterizes the gastric lesions leading to GC. We only found a positive association with IFN-*γ* (Table 2). We found significant ORs and a positive trend for total GC (OR: 3.0; 95% CI: 1.1–8.7), which showed the highest association with intestinal type of GC (OR: 6.4; 95% CI: 1.2–35.4; *p* for trend, *p* = 0.044). No significant associations were found with the other inflammatory mediators: IL-1*β*, IL-6, IL-8, IL-10, TNF-*α*, TGF-*β*, and MCP-1 (Supplementary Table 3). IFN-*γ* is usually secreted by inflammatory T cells and macrophages as part of an active Th1 immune cell response. We classified the GC samples according to the levels of mononuclear (MN) cell infiltration, finding that 59% of samples presented moderate to severe infiltration. However, we did not find significant differences in the levels of IFN-*γ* and the levels of MN cells in the GC samples (Supplementary Figure 2A). We also analyzed 46 patients with nonatrophic gastritis; the mean anti-VCA-IgG antibody for these patients was 85.1 HU/ml with a range of (37.3–202.9). No association was found between the levels of IFN-*γ* and levels of anti-VCA-IgG in this set of patients (Supplementary Table 4). 90% of patients with gastritis samples had mild inflammation (Supplementary Figure 2B). Therefore, in these patients, the levels of inflammation were not associated with the levels of IFN-*γ*.

### 3.4. Analysis of EBV-Positive GC Samples by PCR and Association with Cytokine Levels

**Figure 1:**
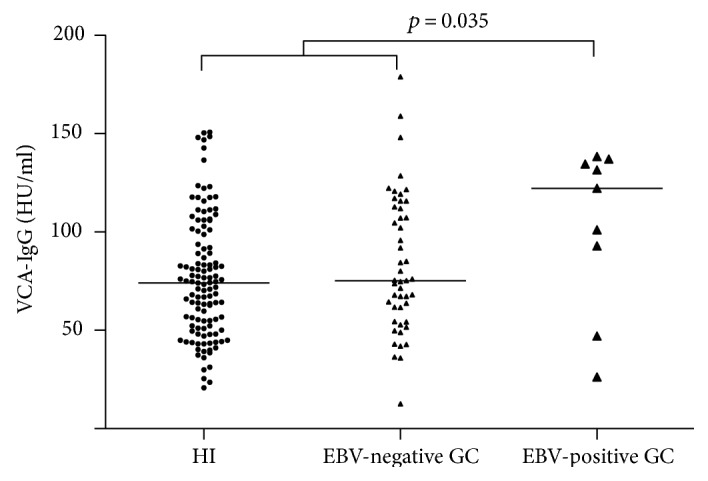
Anti-EBV reactivation antibodies in GC. EBV-positive GC samples were considered after a PCR analysis detecting EBV-positive ≥40,000 viral genomes. Comparisons were made between EBV-positive and EBV-negative GCs and controls. The EBV-positive group presented higher VCA-IgG titers compared with the EBV-negative and control groups (*p* = 0.035). The medians of the VCA-IgG titers were EBV-positive patients (122 HU/ml), EBV-negative (73.4 HU/ml), and HI controls (74.1 HU/ml).

**Figure 2:**
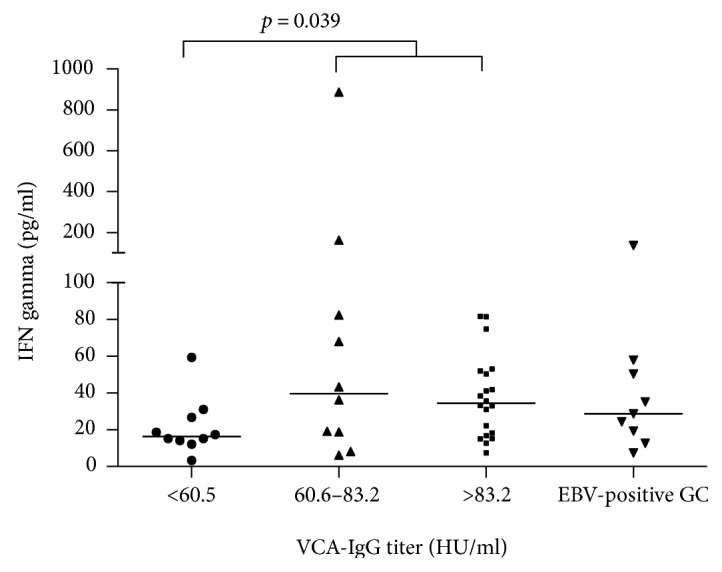
Levels of interferon (IFN) gamma in plasma of patients with intestinal-type GC according to the titer of VCA-IgG antibodies. Two comparisons were made: (1) in which all patients were categorized in tertiles according to the EBV-VCA antibody titers and IFN-*γ* levels were compared in each group and (2) these groups were compared with the selected set of nine patients with EBV PCR-positive GC samples. Note that the EBV PCR-positive samples are also included in the VCA-IgG high group.

We analyzed the presence of EBV genomes in the GC samples using a PCR test previously estimated to detect ≥40,000 viral genomes [22]. This test detects 50-fold of the viral load found in GC tissues in which EBV infection is restricted to B cells [23]; therefore, identifying tissues with a high burden of EBV infection is suggestive of local viral reactivation. Of 97 GC samples from which we could get DNA of good integrity after a *β*-actin PCR test, we found nine positive samples (9.28%). EBV-positive samples were confirmed by a nested PCR [22] and by Sanger sequencing of both strands from the first positive PCR-amplicon (not shown). When we compared the median of the VCA-IgG titers, we found a statistically significant difference between EBV-positive GC samples (122.8 HU/ml) and EBV negative (73.4 HU/ml) samples and HI controls (74.1 HU/ml) (Figure 1). We observed that high IFN-*γ* plasma levels are present in patients clustered either because of an EBV PCR-positive gastric lesion or high VCA-IgG. The EBV PCR-positive group was as high as the 2nd and 3rd group of patients with the highest anti-VCA antibody levels, although without significance perhaps because of the small number of positive samples (Figure 2). Similarly, lower but no significant IL-8 levels were observed in the EBV PCR-positive group, perhaps because of the small number of samples. No differences were observed for IL-1*β* and TNF-*α* (Supplementary Figure 3). However, only three EBV-positive samples were present in this subgroup of 65 GC patients used to analyze these two cytokines.

### 3.5. Analysis of EBV and *HP* Coinfection and Levels of Inflammatory Cytokines

Because infection by *HP* is considered the main risk factor to develop gastric inflammation and GC, and we have previously described a possible cooperative role of EBV [17, 18, 24], we looked for positive associations between *HP* and EBV coinfected patients and the levels of the cytokines. As it has been extensively reported, we found a positive association between *HP* and GC (Table 3). A stronger association was found with diffuse and total GC. We and others have previously observed that anti-*HP* antibodies better correlate with intestinal GC precursor lesions (atrophic gastritis and intestinal metaplasia) [18]. We then divided the *HP*-positive group into two subgroups according to the anti-VCA-IgG antibody titers. Because of the reduced number of patients, we could not use tertiles as before. Of all cytokines, we again only found a significant association with IFN-*γ* (Table 4, and see Supplementary Table 5 for the analysis of the rest of the cytokines and chemokines). Interestingly, the IFN-*γ* association was found for the subtype of diffuse GC and the total GC group (OR: 6.5, 95%, 1.4–30.7 for diffuse and OR: 3.5, 95%, 1.3–9.8 for total GC), suggesting EBV individual responses and in cooperation with *HP* in intestinal and diffuse GCs, respectively. When we repeated this analysis further subdividing the patients in CagA-positive and CagA-negative subgroups, no meaningful associations were found because of the small number of samples (data not shown).

## 4. Discussion

The titer of antibodies against EBV structural proteins has been proposed to correlate with the level of viral reactivation and as a prognostic marker in NPC and EBVaGC [12–16, 25–28]. We have previously reported an association between EBV-VCA reactivation antibodies and the intestinal type of GC [18]. A different cohort of patients from Mexico and Paraguay was analyzed in that study, and the comparison control group was formed of patients with nonatrophic gastritis. In this study, we analyzed a new cohort of 127 patients with GC, which were compared with healthy individuals attending the Medical Center blood bank to donate blood and patients with nonatrophic gastritis. In line with our previous study [18], an association between EBV-VCA and the intestinal type of GC was confirmed. Because intestinal GC progresses through a series of inflammatory lesions, we wanted to know whether EBV may also be a source of the local chronic inflammation that leads to intestinal GC. EBV most studied mechanism of transformation is through expression of its powerful oncogenes, and accordingly, it is classified as a direct transforming infectious agent [29]. Direct transforming agents are part of the genetic lesions that initiate and sustain cancer, as such, they monoclonally infect most tumor cells. There is plenty of evidence supporting EBV direct transformation in EBV-associated malignancies, including lymphomas, nasopharyngeal carcinoma, and about 10% of GCs (the so-called EBVaGCs). Previous studies in GC patients have determined the association between some mediators of inflammation and this disease [11, 30–32]. Chong et al. [30] found that IL-1*β* was the only cytokine that is highly expressed in EBVaGC, and El-Omar et al. [31] found a polymorphism in the *IL-1* gene suspected to enhance cytokine levels, which showed an association with increased risk of GC. Other cytokines that have been reported augmented in GC patients and associated with *HP* and/or anti-EBV-VCA antibodies are IL-8 and TNF-*α* [32]. The association of these cytokines and GC has not been consistent as other groups have not been able to reproduce those observations [21]. In the present study, we searched for associations between VCA-IgG levels and eight mediators of inflammation related either to gastric damage or to an antipathogen response. We could only find a positive association with IFN-*γ*, and no associations were observed with IL-8 and TNF-*α*. Interestingly, this association was only true to the intestinal type of GC but not to the diffuse type.

EBVaGC has been characterized as having a dense intratumoral leukocyte infiltrate, particularly of cytotoxic CD8 positive T cells [33–36]. Because of this characteristic, some studies support that EBVaGC is of good prognosis [35–38]. Besides the cytotoxic activity, activated CD8 positive T cells are characterized by secretion of IFN-*γ*. In future studies, it should be interesting to analyze whether the levels of VCA-IgG closely correlate with clinical parameters of good prognosis.

Strong et al. [11] reported that samples with high levels of EBV gene expression correlated with elevated IFN-*γ* expression. Kim et al. [39] also found high expression of the *IFNG* gene in EBVaGC. Although IFN-*γ* is an important component of the antipathogen immune response, neutralization of this cytokine in mice models of *HP* infection inhibits gastric inflammation [40, 41], supporting a promoting instead of a protective role for IFN-*γ*. Indeed, murine models of gastritis support that Th1 responses enhance disease while Th2 responses are protective [40]. IFN-*γ* has also been observed elevated as early as in pediatric gastric lesions, perhaps in an attempt to protect against *HP* and/or EBV infection [41]. However, it seems that *HP* and EBV have not only found a way to avoid IFN-*γ* but to use this cytokine to counteract immune responses and persist in the gastric mucosa. It has been reported in viral and bacterial coinfections that the presence of IFN-*γ* results in inhibition of macrophage expression of the macrophage receptor with collagenous structure (MARCO), which renders them unable to phagocyte bacteria and virus-infected cells [42]. IFN-*γ* is a strong activator of Th1 T cells, but Strong et al. proposed that cytotoxic T cells and natural killer cells are inhibited by high levels of IDO1 in the stroma of EBVaGCs [11]. Similarly, the TCGA group found elevated levels of IDO1, PD1, and PDL1 in the stroma of EBVaGCs [10]. Interestingly, it was recently reported that IFN-*γ* upregulate PDL-1 expression in human NPC cells [43]. In that study, it was found that levels of serum IFN-*γ* increased along with the EBV DNA load. Multiple studies have also reported an increased expression of PD-L1 in EBVaGC [10, 44–47], and expression of PD-L1 was associated with an IFN-*γ* signature [44]. Although EBV and *HP* may display multiple mechanisms to counteract IFN-*γ* functions in the gastric mucosa, promotion of the antitumor and antipathogen activity of the CD8 T cells with anti-PD-1/PD-L1 antibodies may be a potential therapeutic strategy for EBVaGC. A recent clinical trial with an anti-PD-1 antibody in GC revealed promising antitumoral activity [44].

## 5. Conclusions

We have previously provided evidence of an association between the levels of VCA-IgG and the intestinal type of GC [19]. We confirm that association in this study. Because chronic inflammation is critical to drive intestinal GC and a few cytokines and chemokines have previously been associated with gastric inflammation, in this study, we address whether the systemic levels of IL-1*β*, IL-6, IL-8, IL-10, TNF-*α*, TGF-*β*, MCP-1, and IFN-*γ* also correlated with VCA-IgG, which could further support a role for EBV in gastric inflammation. We only found evidence of a positive association with IFN-*γ*. Although this cytokine is an important component of protective antiviral immune responses, it can have both pro- and anti-inflammatory effects, with several studies supporting that IFN-*γ* contributes to GC progression. Presently, it is unclear whether the increased levels of IFN-*γ* are reflecting an increased immune response aiming to restrain EBV activity or the cytokine is contributing with *HP*/EBV-induced GC presentation.

One limitation of the study is that five of the cytokines studied (IL-1*β*, TNF-*α*, IL-6, IL-10, and MCP-1) could only be tested in a subset of 65 GC patients. It is possible that other significant associations could be evidenced with a higher number of patients. Also, we were unable to observe any association with *HP* nor CagA and this may also be related to the number of samples tested. Here is important to mention that the ELISA test used in the present study has a sensitivity of 85% and a specificity of 87% [21]; therefore, it is possible to have false negative samples.

## Supplementary Material

Supplementary Figure 1. Age distribution of patients with intestinal type GC and according to the titer of EBV reactivation antibodies. No statistical differences were found, with the three groups presenting similar average age. Supplementary Figure 2. Levels of Interferon gamma (INF-?) in plasma of patients with GC and in gastritis according to the levels of mononuclear (MN) cell infiltration. Patients were categorized in mild, moderate and severe MN infiltrate. A) The GC samples presented 59% of moderate to severe infiltrationB) 90% of patient's gastritis present mild inflammation. . No significant differences in the INF-? levels of MN cell in the GC and gastritis patients. Supplementary Figure 3. Systemic levels of IL-8, IL-1β and TNF-a. Comparisons were made between the patients with intestinal and diffuse GCs, the healthy controls and the EBV positive group by PCR. Although no statistical differences were found, lower levels of IL-8 are clearly observed for IL-8 in the EBV positive group. Medians (pg/ml): HI = 19, Intestinal = 18, Diffuse = 21 and EBVpos GC = 5.3. p > 0.1 for all comparisons. Supplementary table S1. Number of cases included for the different cytokines and chemokines. Supplementary Table S2. General description of the study population and association of EBV VCA-IgG titers with GC. Supplementary Table S3. OR according to VCA-IgG titers and cytokine levels. Supplementary Table 4. ORs of VCA-IgG and INF-? levels in gastritis patients. Supplementary Table S5. ORs according to the VCA-IgG antibody titers, HP positivity and cytokine levels.

## Figures and Tables

**Table 1:** Description of the study population and association of EBV antibody titers with GC.

Variable	Healthy controls^a^	Gastric cancer							
		Intestinal		Diffuse		Mixed		Total gastric cancer	
Number studied (%)	118 (100)	40 (31.5)		63 (49.6)		24 (18.9)		127 (100)	
Age (mean ± S.D.)^b^	34.6 ± 11.3	67.1 ± 12.3^∗∗^		57.7 ± 13.2^∗∗^		63.7 ± 13.3^∗∗^		61.8 ± 13.5^∗∗^	
Sex, male/female ratio	48/70 = 0.68	23/17 = 1.4		38/25 = 1.5		12/12 = 1		73/54 1.35	
VCA-IgG titer^c,d^ (median) range	74.1 (23.5-150.4)	**90.5** ^e^ (24.9–148)		73.5 (25.2–179)		82.3 (35.9–141.6)		76.0 (24.9–179)	
VCA-IgG titer^c^	*n*	*n*	OR (95% CI)	*n*	OR (95% CI)	*n*	OR (95% CI)	*n*	OR (95% CI)
23.5–60.5	39	9	1.0	22	1.0	6	1.0	37	1.0
60.6–83.2	40	10	1.2 (0.3–5.3)	16	0.9 (0.4–2.6)	6	1.9 (0.4–9.3)	32	1.0 (0.4–2.5)
83.3–150.7	39	21	3.0 (0.7–12.6)	25	0.6 (0.2–1.9)	12	3.2 (0.7–15.7)	58	0.8 (0.3–2.2)
*p* for trend			0.047^∗^		0.714		0.176		0.122

^a^Population included for the analysis of IL-8, IFN-*γ*, and TGF-*β*. ^b^Student's *t*-test. ^c^HU·ml^−1^. ^d^Only positive values were considered. ^e^*p* < 0.073. ^∗^*p* < 0.05, ^∗∗^*p* < 0.01.

**Table 2:** ORs of EBV titers and IFN-*γ* levels.

Variable	Healthy controls			Gastric cancer											
				Intestinal			Diffuse			Mixed			Total GC		
VCA-IgG titers^a^	*n* low IFN-*γ*	*n* high IFN-*γ*	OR (95% CI)	*n* low IFN-*γ*	*n* high IFN-*γ*	OR (95% CI)	*n* low IFN-*γ*	*n* high IFN-*γ*	OR (95% CI)	*n* low IFN-*γ*	*n* high IFN-*γ*	OR (95% CI)	*n* low IFN-*γ*	*n* high IFN-*γ*	OR (95% CI)
23.5–60.5	22	17	1.0	6	3	1.0	10	12	1.0	1	5	1.0	17	20	1.0
60.6–83.2	27	13	0.7 (0.4–1.3)	2	8	**8.0 (1.0–63.9)**	3	13	3.6 (0.8–16.3)	2	4	0.4 (0.1–6.2)	7	25	**3.0 (1.1–8.7)**
83.3–150.7	15	24	1.4 (0.9–2.2)	5	16	**6.4 (1.2–35.4)**	8	17	1.7 (0.5–5.8)	3	9	0.6 (0.1–7.4)	16	42	2.2 (0.9–5.3)
*p* for trend			0.113			**0.044** ^∗^			0.356			0.781			0.088

^a^HU·ml^−1^; ^∗^*p* < 0.05.

**Table 3:** Analysis of the *HP* status in the gastric cancer patients.

Variable	Healthy controls^a^	Gastric cancer			
		Intestinal	Diffuse	Mixed	Total GC
*n*	118	40	63	24	127
*HP*					
Positive, *n* (%)	58 (49.2)	22 (55)	49 (77.8)	17 (70.8)	88 (69.3)
*p* ^b^		0.643	**0.002**	0.116	**0.015**
*CagA+*					
Positive, *n* (%)	35 (29.7)	14 (35)	38 (60.3)	13 (54.2)	65 (73.9)
*p* ^b^		0.718	**0.009**	0.117	**0.000**

CagA = cytotoxin-associated gene A; *HP* = *Helicobacter pylori*. Numbers in bold denote statistical significance (*p* < 0.05). ^a^Used as control group; ^b^proportion test for positive samples.

**Table 4:** ORs according to the VCA-IgG titers and the status of IFN-*γ* in *HP*-positive individuals.

Variable	Healthy controls			Gastric cancer											
				Intestinal			Diffuse			Mixed			Total GC		
VCA-IgG titers^a^	*n* Neg IFN-*γ*	*n* Pos IFN-*γ*	OR (95% CI)	*n* Neg IFN-*γ*	*n* Pos IFN-*γ*	OR (95% CI)	*n* Neg IFN-*γ*	*n* Pos IFN-*γ*	OR (95% CI)	*n* Neg IFN-*γ*	*n* Pos IFN-*γ*	OR (95% CI)	*n* Neg IFN-*γ*	*n* Pos IFN-*γ*	OR (95% CI)
23.5–60.5	12	7	1.0	4	3	1.0	9	6	1.0	1	4	1.0	14	13	1.0
60.6–150.7	21	18	1.5 (0.5–4.7)	3	12	6.3 (0.7–56.7)	10	24	**6.5 (1.4–30.7)**	4	8	0.5 (0.1–7.6)	17	44	**3.5 (1.3–9.8)**

^a^HU·ml^−1^. Numbers in bold denote positive associations. *HP* = *Helicobacter pylori*.
