# Supplementary material for: Elevated Levels of Interferon-γ Are Associated with High Levels of Epstein-Barr Virus Reactivation in Patients with the Intestinal Type of Gastric Cancer
Source: J Immunol Res. 2017 Nov 14;2017:7069242. doi: 10.1155/2017/7069242 (PMC5733903; doi:10.1155/2017/7069242)

Supplementary Figure 1. Age distribution of patients with intestinal type GC and according to the titer of EBV reactivation antibodies. No statistical differences were found, with the three groups presenting similar average age.

Supplementary Figure 2. Levels of Interferon gamma (INF-γ) in plasma of patients with GC and in gastritis according to the levels of mononuclear (MN) cell infiltration. Patients were categorized in mild, moderate and severe MN infiltrate. A) The GC samples presented 59% of moderate to severe infiltrationB) 90% of patient's gastritis present mild inflammation. . No significant differences in the INF-γ levels of MN cell in the GC and gastritis patients.

Supplementary Figure 3. Systemic levels of IL-8, IL-1β and TNF-α. Comparisons were made between the patients with intestinal and diffuse GCs, the healthy controls and the EBV positive group by PCR. Although no statistical differences were found, lower levels of IL-8 are clearly observed for IL-8 in the EBV positive group. Medians (pg/ml): HI = 19, Intestinal = 18, Diffuse = 21 and EBVpos GC = 5.3. $p > 0.1$ for all comparisons.

Supplementary table S1. Number of cases included for the different cytokines and chemokines

	Part 1<br>No. tested for IL-8, IFN-γ and TGF-β<br><br>Plasma	Part 2<br>No. tested for IL-1β, TNF-α, IL-6, IL-10 and MCP-1<br><br>Serum
Healthy donors	118	113
Gastric cancer	127	65
Total	235	178

Supplementary Table S2. General description of the study population and association of EBV VCA-IgG titers with GC.

Variable	Healthy Controls[a]	Gastric Cancer							
		Intestinal		Diffuse		Mixed		Total Gastric Cancer	
Number studied (%)	197 (100)	40 (31.5)		63 (49.6)		24 (18.9)		127 (100)	
Age (mean ± s. d.)[b]	35.0 ± 10.9	67.1 ± 12.3**		57.7 ± 13.2**		63.7 ± 13.3**		61.8 ± 13.5**	
Sex, male/ Female ratio	98/99= 0.99	23/17 = 1.4		38/25= 1.5		12/12=1		73/54 1.35	
VCA-IgG titer[c,d] (median)<br>Range	75.1<br>(20.7-160.7)	**90.5**[e]<br>(24.9-148)		73.5<br>(25.2-179)		82.3<br>(35.9-141.6)		76.0<br>(24.9-179)	
VCA-IgG titer[c]	n	n	0R (95% CI)	n	0R (95% CI)	n	0R (95% CI)	n	0R (95% CI)
20.7 -59.7	65	9	1.0	22	1.0	6	1.0	37	1.0
59.8 -85.5	66	10	1.8 (0.5-6.8)	18	1.3 (0.5- 2.9)	7	0.98 (0.3-3.3)	35	1.3 (0.6-2.9)
85.6-160.7	66	21	**3.8 (1.1 -13.7)***	23	0.9 (0.3-2.3)	11	3.7 (0.9- 15.6)	55	1.1 (0.5-2.6)
p for trend			**0.039***		0.928		0.251		0.147

[a]Analysis including the 197 HI samples
[b]Student's *t*-test
[c]HU ml-1
[d]Only positive values were considered
[e]$p < 0.073$
*$p < 0.05$, **$p < 0.01$

Supplementary Table S3. OR according to VCA-IgG titers and cytokine levels.

Variable	Healthy Controls	Gastric Cancer			
		Intestinal	Diffuse	Mixed	Total GC
	OR (95% CI)	OR (95% CI)	OR (95% CI)	OR (95% CI)	OR (95% CI)
VCA-IgG titers[a]	**TGFβ**				
23.5 -60.5	1.0	1.0	1.0	1.0	1.0
60.6 -83.2	1.1 (0.8-1.6)	1.3 (0.2-10.5)	1.4 (0.4-5.2)	1.0 (0.1-11.0)	1.1 (0.6-1.9)
83.3-150.7	0.9 (0.5-1.3)	2.3 (0.4-13.9)	1.0 (0.3-3.2)	0.4 (0.1-3.9)	1.3 (0.8-2.1)
p for trend	0.498	0.334	1.0	0.403	0.367
	**IL-8**				
23.5 -60.5	1.0	1.0	1.0	1.0	1.0
60.6 -83.2	1.3 (0.8-2.3)	2.7 (0.7-10.1)	1.1 (0.3-1.9)	0.3 (0.5-2.3)	1.1 (0.7-1.9)
83.3-150.7	1.1 (0.7-2.0)	2.4 (0.6-8.6)	0.9 (0.6-1.7)	0.8 (0.3-2.3)	1.1 (0.7-1.8)
p for trend	0.648	0.204	0.969	0.901	0.623
	**IL-1β**				
23.5 -60.5	1.0	1.0	1.0	1.0	1.0
60.6 -83.2	0.5 (0.2-1.3)	-	-	-	-
83.3-150.7	0.6 (0.2-1.3)	-	5.0 (0.7-35.5)	4.0 (0.2-75.7)	2.2 (0.6-7.6)
p for trend	0.208	0.071	0.224	0.361	0.447
	**MCP-1**				
23.5 -60.5	1.0	1.0	1.0	1.0	1.0
60.6 -83.2	1.2 (0.5-3.2)	1.0 (0.1-16)	4.8 (0.4-58.0)	1.0 (0.1-29.8)	1.8 (0.4-7.7)
83.3-150.7	0.9 (0.4-2.1)	1.0 (0.1-9.2)	4.0 (0.4-42.2)	1.5 (0.1-21.3)	1.9 (0.5-6.7)
p for trend	0.732	1.0	0.320	0.771	0.354
	**TNFα**				
23.5 -60.5	1.0	1.0	1.0	1.0	1.0
60.6 -83.2	1.8 (0.6-5.4)	1.0 (0.1-24.5)	2.6 (0.3-22.9)	-	2.0 (0.4-10.5)
83.3-150.7	0.95 (0.4-2.4)	0.9 (0.1-11.6)	2.0 (0.3-13.6)	0.5 (0.3-8.9)	1.3 (0.3-4.6)
p for trend	0.887	0.938	0.521	0.602	0.857
	**IL-10**				
23.5 -60.5	1.0	1.0	1.0	1.0	1.0
60.6 -83.2	1.4 (0.4-4.5)	-	0.7 (0.1-5.1)	3.0 (0.1-107)	1.4 (0.3-6.6)
83.3-150.7	1.2 (0.4-3.5)	-	0.9 (0.1-5.5)	4.5 (0.3 -80	2.3 (0.6-8.7)
p for trend	0.789	0.079	0.965	0.322	0.185
	**IL-6**				
23.5 -60.5	1.0	1.0	1.0	1.0	1.0
60.6 -83.2	0.6 (0.1-7.4)	1.0 (0.1-16)	4.8 (0.4-58.0)	3.0 (0.1-107)	2.4 (0.5-11.1)
83.3-150.7	0.4 (0.4-4.9)	0.7 (0.1-6.11)	4.0 (0.4-42.2)	2.0 (0.1-35.8)	1.8 (0.5-6.9)
p for trend	0.486	0.677	0.319	0.672	0.477

[a]HU ml-1

Supplementary Table 4. ORs of VCA-IgG and INF-γ levels in gastritis patients.

**Variable**	**Gastritis**		
VCA-IgG titers[a]	n Neg INF γ	n Pos INF γ	OR (95% CI)
23.5 -60.5	4	9	1.0
60.6 -83.2	1	8	1.3 (0.8-2.0)
83.3-150.7	4	20	1.2 (0.8-1.8)
p for trend			0.360

Supplementary Table S5. ORs according to the VCA-IgG antibody titers, *HP* positivity and cytokine levels.

Variable	Healthy Controls	Gastric Cancer			
		Intestinal	Diffuse	Mixed	Total GC
VCA-IgG titers[a]	OR (95% CI)	OR (95% CI)	OR (95% CI)	OR (95% CI)	OR (95% CI)
	**TGF-β**				
23.5 -60.5	1.0	1.0	1.0	1.0	1.0
60.6 -150.7	0.9 (0.3-2.9)	1.5 (0.2-12.9)	0.8 (0.2-02.8)	0.1 (0.1-11.7)	1.04 (0.4-2.7)
	**IL-8**				
23.5 -60.5	1.0	1.0	1.0	1.0	1.0
60.6 -150.7	1.3 (0.4-4.0)	10.4 (0.6-168)	0.9 (0.2-3.6)	0.6 (0.1-6.6)	1.2 (0.5-3.2)
	**IL-1β**				
23.5 -60.5	1.0	1.0	1.0	1.0	1.0
60.6 -150.7	0.9 (0.3-2.8)	-	1.2 (0.1-14.5)	0.1 (0.1-40.6)	2.1 (0.4-10.6)
	**IL-6**				
23.5 -60.5	1.0	1.0	1.0	1.0	1.0
60.6 -150.7	-	0.8 (0.1-12.2)	1.3 (0.1-16.4)	1.0 (0.01-139)	0.9 (0.2-3.8)
	**IL-10**				
23.5 -60.5	1.0	1.0	1.0	1.0	1.0
60.6 -150.7	0.5 (0.1-1.8)	-	0.4 (0.1-5.7)	1.1 (0.1-98.9)	1.3 (0.3-6.3)
	**MCP-1**				
23.5 -60.5	1.0	1.0	1.0	1.0	1.0
60.6 -150.7	0.9 (0.3-2.8)	0.8 (0.5-12.5)	2.3 (0.2-33.1)	0.5 (0.1-12.5)	0.9 (0.2-3.6)
	**TNF-α**				
23.5 -60.5	1.0	1.0	1.0	1.0	1.0
60.6 -150.7	2.1 (0.7-5.9)	0.5 (0.03-8.6)	2.5 (0.3-25.3)	2.2 (0.1-97.8)	1.2 (0.3-5.5)

[a]HU ml-1

SUPPLEMENTARY FIGURE 1

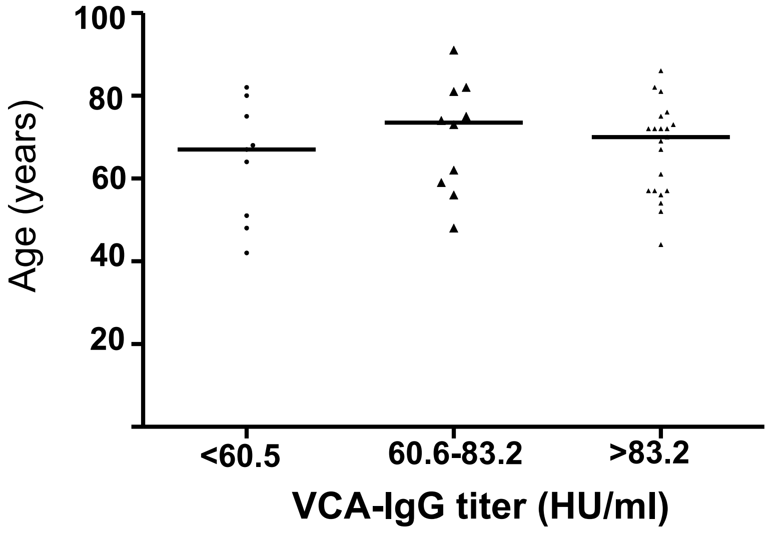

SUPPLEMENTARY FIGURE 2

A)

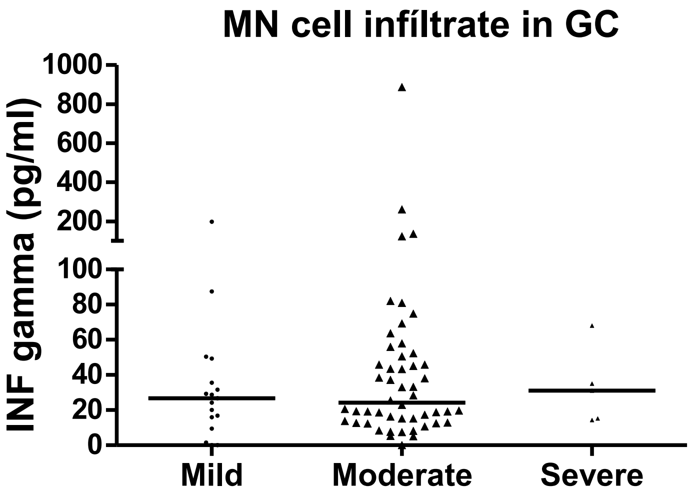

B)

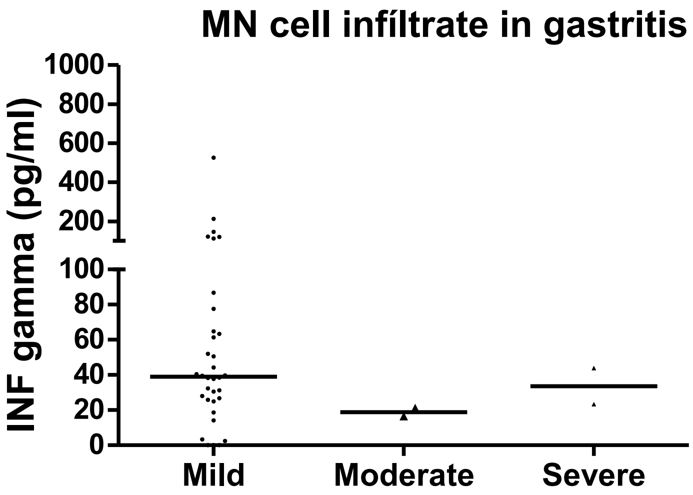

SUPPLEMENTARY FIGURE 3

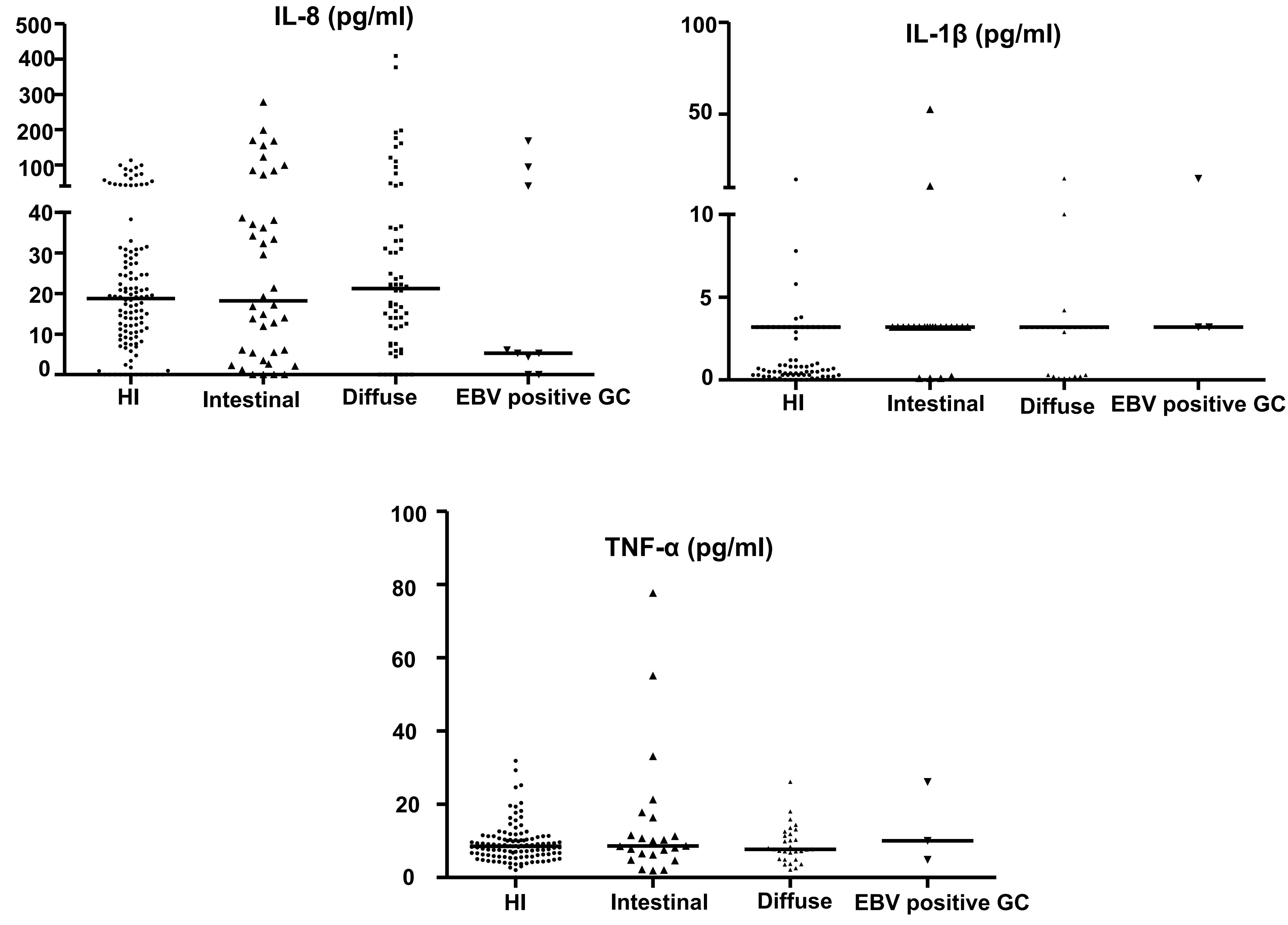

Supplement: Supplementary file 1 — Supplementary Figure 1. Age distribution of patients with intestinal type GC and according to the titer of EBV reactivation antibodies. No statistical differences were found, with the three groups presenting similar average age. Supplementary Figure 2. Levels of Interferon gamma (INF-?) in plasma of patients with GC and in gastritis according to the levels of mononuclear (MN) cell infiltration. Patients were categorized in mild, moderate and severe MN infiltrate. A) The GC samples presented 59% of moderate to severe infiltrationB) 90% of patient's gastritis present mild inflammation. . No significant differences in the INF-? levels of MN cell in the GC and gastritis patients. Supplementary Figure 3. Systemic levels of IL-8, IL-1β and TNF-a. Comparisons were made between the patients with intestinal and diffuse GCs, the healthy controls and the EBV positive group by PCR. Although no statistical differences were found, lower levels of IL-8 are clearly observed for IL-8 in the EBV positive group. Medians (pg/ml): HI = 19, Intestinal = 18, Diffuse = 21 and EBVpos GC = 5.3. p > 0.1 for all comparisons. Supplementary table S1. Number of cases included for the different cytokines and chemokines. Supplementary Table S2. General description of the study population and association of EBV VCA-IgG titers with GC. Supplementary Table S3. OR according to VCA-IgG titers and cytokine levels. Supplementary Table 4. ORs of VCA-IgG and INF-? levels in gastritis patients. Supplementary Table S5. ORs according to the VCA-IgG antibody titers, HP positivity and cytokine levels. [file 7069242.f1.pdf]
